# Development and physicochemical characteristics of multicomponent freeze-dried snacks obtained with blackcurrant pomace powder and calcium ions as structuring agents

**DOI:** 10.1007/s13197-023-05906-w

**Published:** 2024-01-22

**Authors:** Magdalena Karwacka, Sabina Galus, Monika Janowicz

**Affiliations:** Department of Food Engineering and Process Management, Institute of Food Sciences, Warsaw University of Life Sciences, SGGW, 159c Nowoursynowska St, 02-787 Warsaw, Poland

**Keywords:** Blackcurrant pomace, Calcium ions, Low-methoxyl pectin, Freeze-dried snacks, Physicochemical properties, Microstructure

## Abstract

Consumers seek healthy and sustainable products, whereas the food industry faces the challenge of processing by-products management. The application of fruit pomace as an additive could be a solution addressing the needs of both consumers and producers. The research objective has been to assess the effect of dried blackcurrant pomace powder (BP) and calcium ions in varied concentration on the physicochemical properties of multicomponent freeze-dried snacks as compared to the influence of low-methoxyl pectin (LMP). The snacks were prepared using varied content of BP (1, 3, 5%) and calcium lactate (0, 0.01, 0.05%). Water content and activity, hygroscopic properties, structure, texture, colour, polyphenols content (TPC), and antioxidant activity were analysed. The addition of BP resulted in lowering water activity and porosity. The microstructure of the snacks consisted of a large number of small and unevenly distributed pores. Consequently, the reduction of hygroscopic properties with the growing amount of BP was observed. Applied additives strengthened the structure and caused changes in compression curves indicating enhanced hardness and crispiness. The effect given by 5% of BP was comparable to that obtained with 0.5% of LMP. Additionally, blackcurrant pomace infusion increased TPC and enhanced antioxidant activity but it also caused significant changes in the colour of the snacks. Overall, obtained results have shown that dried blackcurrant pomace powder (BP) can be successfully applied as a food additive supporting stability, texture, and bioactive compounds content, thus fortifying the physicochemical properties of freeze-dried fruit and vegetable snacks.

## Introduction

Snacks are products that attract consumers through their textural and sensory attributes (Silva-Espinoza et al. 2021b). They are designed to be consumed between the main meals or as potential replacements for them. Nowadays, when consumers’ consciousness has notably improved in respect of nutrition and environmental concerns, producers are obliged to face their expectations by introducing high-quality, healthy, and sustainable products alternating sweets and high-fat snacks available in the food market (Ciurzyńska et al. 2019).

Food waste management is a global issue usually dealt with in consideration of food products that either spoil before being consumed or are discarded because of expiration (Tomaszewska et al. 2022). However, industrial waste and by-products are not even accounted for as food waste, and they are not included in the definition of consumable products consciously discarded at any phase of a life cycle. Organic matter may be recycled and used for other purposes, such as animal feed or a source of precious compounds that the industry is capable of separating (Garcia-Garcia et al. 2019).

The objective of this research has been set out developed in relation to the feasibility of manufacturing healthy snacks that align with the zero waste and environmentally friendly trends. To address the nutritional aspect, freeze-drying has been selected as the preservation method. It is significant to ensure high retention of bioactive compounds, achievable through reduced pressure that is effective at much lower temperature as compared to other dehydration methods, e.g. convective drying. Due to the limited exposure to air and increased temperature, adverse impact, such as antioxidant degradation, is reduced to the minimum (Nowak and Jakubczyk 2020). Moreover, freeze-drying provides products that maintain the original shape and are characterised by attractive texture and high porosity, which leads to improvement in active components’ bioavailability (Ciurzyńska et al. 2015; Pollini et al. 2022).

Due to the quality of given products, freeze-drying had been previously applied in the course of the production of multicomponent snacks based on hydrocolloid gels (pectin, sodium alginate, gums, starches, and fibers) with the addition of fruit and vegetables, purees, concentrates, juices, etc. (Ciurzyńska et al. 2015, 2020; Silva-Espinoza et al. 2020a; Jakubczyk et al. 2022). Although a lot of research has been carried out on this topic in the last decade, it had been mostly focused on the physical characteristics of the products. The access to the information on the nutritional value or consumer acceptance of snacks made of freeze-dried gels is very limited to this day, and only a few papers are available. Existing studies show that this type of snack is evaluated on the basis of personal preferences, attributes related to flavour, texture and colour, emphasized by consumers (Silva-Espinoza et al. 2021b). However, the high porosity and hygroscopic nature of freeze-dried gels necessitate the selection of proper packaging to prevent the quality loss, especially texture changes occurring due to moisture absorption (Silva-Espinoza et al. 2020b). Moreover, the use of biopolymers enhances not only structural and textural parameters but also increases fibre content (Karwacka et al. 2022c) and assures protection of the bioactive compounds, such as vitamin C and polyphenols, during digestion (Silva-Espinoza et al. 2021a).

Based on available research, pectin that a plant-derived high molecular weight polysaccharide is one of the structuring agents used for developing freeze-dried gel-based snacks (Ciurzyńska et al. 2015; Hu et al. 2022). Functional properties of pectin vary depending on the origin and chemical structure, thus it is usually applied in products characterised by relatively high water content as gelling, thickening, stabilising, or emulsifying additive (Lara-Espinoza et al. 2018). However, as the previously mentioned authors have already established, after dehydration, freeze-dried pectin gels feature a highly porous structure that accounts for a high rehydration rate, low shrinkage during processing as well as attractive texture, acoustic, and sensory properties. Pectin is commonly extracted from fruit pomace that is a solid residue left after juice processing. Addressing the environmental aspect of consumers’ expectations, this research has been designed to examine the possibility of substituting a traditional structuring agent with the material of its origin.

Given that Poland is one of the major blackcurrant producers, and the berries are cultivated mostly for the purpose of producing juice (60%) and jam, the amount of generated residue is significant, therefore it is necessary to explore new ways to manage it in the plausibly most effective way (Kraciński 2014; Cortez and Mejia 2019). According to the estimates put forward by Poland’s Central Statistical Office, the cultivation area of currant has been constantly growing, and so have the blackcurrant crops. In 2021 the production output was estimated to equal 114 800 tons and the preliminary assessment indicates that in 2022 the overall production output arising from fruit bushes and berry plantations may have posted a year-on-year increase of about 9.3% (Statistics Poland 2022). Blackcurrant pomace demonstrates high nutritional and health-promoting value thanks to high bioactive compounds and fibre content (Xue et al. 2022). Blackcurrant is also rich in pectin, and has been comprehensively characterised in the recent report by (Pancerz et al. 2022). It is usually disposed of as nutritious animal feed, a raw material for bioactive compounds and colorants extraction. But pomace has been recently studied in terms of an ingredient fortifying the functional properties of several food products, mostly bakery and confectionery (Majerska et al. 2019). As a consequence of all of the above, blackcurrant pomace powder has been chosen as the research input for the purpose of this study.

The gelation of pectin is determined by its physicochemical characteristics as well as environmental conditions, such as pH, the presence of sugars and proteins, and ions inducing the formation of a three-dimensional network of pectin gels (Chan et al. 2017). Combining these determinants, the latest reports on the risk of calcium deficiency in the population (Shlisky et al. 2022), and the fact that using pomace powder pectin is captured in the particles that could limit its gelling ability, Ca^2+^ ions have been incorporated to potentially support the gelation process and improve the nutritional value of the obtained products.

The purpose of the study has been to analyse the effect of blackcurrant pomace powder and calcium ions addition in varied concentration on the physicochemical quality of multicomponent freeze-dried snacks as compared to snacks structured with pectin.

## Materials and methods

### Materials

The research material was freeze-dried carrot-orange-ginger (COG) snacks obtained by means of dried blackcurrant pomace powder (BP) and low-methoxyl pectin (LMP) used separately as structuring agents. Samples were prepared using frozen carrot cubes supplied by Unifreeze sp. z o.o. (Poland), orange juice concentrate (Purena, Poland), ginger (local market, Warsaw, Poland), dried blackcurrant pomace (Greenherb, Poland), low-methoxyl pectin (Hortimex, Poland), and calcium lactate (Agnex, Poland). Chemicals and reagents were purchased from POCH (Poland) and stored in a dark refrigerator at 4 °C.

### Methods

#### Sample preparation

The samples were prepared according to the methodology developed by Karwacka et al. (2022) and formulations presented in Table 1. The basic formulation of snacks consisted of frozen carrots, orange juice concentrate, and ginger in the same proportion for all samples. The varied additives, blackcurrant pomace powder, pectin and calcium lactate, were added as shown in Table 1.

**Table 1:** Formulation of the multicomponent, carrot-orange-ginger snacks obtained with blackcurrant pomace powder (BP) and low-methoxyl pectin (LMP).

Sample	Carrot	Orange juice concentrate	Ginger	Water	Blackcurrant pomace powder	Pectin	Calcium lactate
	g/100 g						
COG	60	10	0.40	29.60	–	–	–
COG-BP1a	60	10	0.40	28.60	1	–	0
COG-BP1b	60	10	0.40	28.59	1	–	0.01
COG-BP1c	60	10	0.40	28.55	1	–	0.05
COG-BP3a	60	10	0.40	26.60	3	–	0
COG-BP3b	60	10	0.40	26.59	3		0.01
COG-BP3c	60	10	0.40	26.55	3	–	0.05
COG-BP5a	60	10	0.40	24.60	5	–	0
COG-BP5b	60	10	0.40	24.59	5	–	0.01
COG-BP5c	60	10	0.40	24.55	5	–	0.05
COG-LMP0.5b	60	10	0.40	29.09	–	0.5	0.01
COG-LMP1.5b	60	10	0.40	28.09	–	1.5	0.01

Carrot had thawed at 25 °C for up to an hour before it was processed. The LMP and BP were hydrated in calcium lactate solution at 85 °C for 1 min. Then all components were blended in the laboratory knife mill GRINDOMIX GM 200 (Retsch, Germany) at 4500 rpm for 1 min. The prepared mixture was poured into silicone molds, frozen at − 40 °C for about 4 h, and freeze-dried in Alpha 1–2 LD plus freeze-dryer (Martin Christ GmbH, Germany) at 30 °C and the pressure of 0.063 kPa for about 48 h. Prior to testing, snacks were packed in the high-barrier laminate (PET/AI/PE) packaging impermeable to light, gas, and vapour and stored for 48 h at room temperature (25 ± 1 °C).

### Analytical methods

#### Water content and activity

Water content (WC) was obtained by means of the oven method at 70 °C for 24 h (Wiktor et al. 2022). Water activity (*a*_*w*_) was measured using HygroLab C1 (Rotronic, Switzerland) at 25 ± 1 °C. Both analyses were made in triplicate.

#### Hygroscopicity determination

Hygroscopic properties were determined by exposing the sample to a humid environment (RH = 75%) in a desiccator filled with saturated NaCl solution for 72 h at 25 ± 1 °C. The samples had been periodically weighted after 0.5, 1, 3, 6, 9, 12, 24, 48, and 72 h until their weight was constant (Karwacka et al. 2022b). The test was performed in triplicate for each respective sample.

#### Mechanical properties

The sample mechanical properties were measured using TA.HD plus texture analyser (Stable Micro Systems, UK). The samples were compressed using a 20 mm diameter platen probe moving at a speed of 0.5 mm/s. The test had been performed in 10 repetitions until 50% deformation of the initial height of the material (1.5 × 1.5 × 1.5 cm) was obtained (Karwacka et al. 2021). Hardness and compression curves were determined.

#### Microstructure and porosity analysis

The porosity and microstructure were analysed using the X-ray micro-CT SkyScan 1272 system (Bruker microCT, Belgium) with dedicated software (NRecon1.6.3.2, Bruker microCT), directly following the method described previously (Karwacka et al. 2021).

#### Colour determination

Colour parameters L*a*b* were determined by means of CR-5 Colorimeter (Konica Minolta, Japan) with an 8 mm measuring hole and reflectance mode. The measurements were performed in 15 repetitions. The total colour difference (*ΔE*) was calculated according to the equation formula (Hu et al. 2022):

$$\varDelta E=\sqrt{{\left({\varDelta L}^{*}\right)}^{2}+{\left({\varDelta a}^{*}\right)}^{2}+{\left({\varDelta b}^{*}\right)}^{2}}$$

where ${\varDelta L}^{*}$, ${\varDelta a}^{*}$, ${\varDelta b}^{*}$ are the differences in lightness (L*), redness (a*) and yellowness (b*) between the COG snack and samples with additives.

#### Chemical properties analysis

##### Extraction procedure

The chemical analysis of the snacks, including the sample preparation, total phenolic content and antioxidant activity determinations, was carried out according to the methodology presented by Wiktor et al. (2022) with slight modifications. The extracts subjected to the further analysis were prepared by means of extracting 0.3 g of the ground material in 10 mL of 80% (v/v) aqueous ethanol solution for 24 h at ambient temperature and concurrent continuous stirring on a shaker (Heidolph Instruments, Germany) at 1700 rpm. After that, the extracts were centrifuged for 2 min at 3000 rpm in a laboratory centrifuge (MegaStar 600, VWR, Belgium). Each sample was extracted twice.

##### Total phenolic content (TPC) determination

The TPC determination was conducted by means of the spectrophotometric method with Folin-Ciocalteau’s reagent. The extracts were prepared for the analysis purposes by diluting 10 µL in 96-well plates with distilled water (1:1 v/v). Next, 40 µL of 5-fold diluted Folin-Ciocalteau’s reagent was added, mixed, and incubated at ambient temperature for 3 min. To stop the reaction, 250 µL of 7% sodium carbonate solution was infused, and then the mixtures were incubated again in a dark place for 60 min. A blank test was performed by replacing an extract with the extraction reagent. The absorbance was measured using a plate reader (Multiskan Sky, Thermo Electron Co., USA) at a wavelength of 750 nm. The results were expressed in terms of milligrams of gallic acid equivalent per 1 g of dry matter of the sample (mg GAE/g d.m.). The analysis was performed in triplicate for each extract.

##### Antioxidant activity

The ABTS^●+^ assay proceeded by dispensing 10 µL of the analyte and 250 µL of the free radical solution with absorbance at a wavelength of 734 nm 0.7 ± 0.02 onto a 96-well plate, shaking and incubating in a dark place for 6 min. A blank probe was prepared using 80% (v/v) ethanol instead of the extract. The absorbance was measured at a wavelength of 734 nm by means of a Multiskan Sky plate reader. The DPPH^●^ assay was performed similarly to the procedure described for ABTS^●+^ but the incubation lasted for 30 min and the absorbance was measured at 515 nm. The antiradical activity was calculated as the decrease in the absorbance of the radical solution in the presence of an analyte extract and expressed in terms of mg Trolox/g of dried material. This procedure was carried out in triplicate for each extract.

### Statistical analysis

Significant differences between obtained results were evaluated by means of the one-way ANOVA and a post-hoc Tukey’s test at *P* < 0.05. The statistical analysis was carried out using STATISTICA 13 software (TIBCO Software, USA).

## Results and discussion

### Water content, water activity, and hygroscopicity

Apart from the effect hydrocolloids have on structure and texture, these additives were used for reducing water activity thus prolonging the stability of the products (Silva-Espinoza et al. 2020a). The freeze-dried snacks were characterised in terms of water content and water activity, the results of which are shown in Table 2. As expected, the addition of low-methoxyl pectin significantly decreased the water activity of the samples and such an outcome was obtained without diminution in water content. The infusion of blackcurrant pomace instead of LMP also induced *a*_*w*_ lowering but it was linked to the water content decrease. What can be clearly seen in water content and activity results is the pattern related to the increasing amount of BP in the formulation. Such observations indicate that LMP might have interacted with the water molecules in a way that reduced their availability for chemical reactions or microbial growth, even though the total water content remained higher as compared to other samples. The lowering effect of BP on water activity was connected to the decrease in the actual amount of water content. This suggests that the effect of blackcurrant pomace on reducing water activity might be related to its ability to absorb or hold water, leading to the decrease in the overall water content in the samples. Both parameters were remarkably lower for snacks with pomace at 1%, and then 3%, but the statistical analysis did not find any difference between samples with 3 and 5% of BP powder. However, higher blackcurrant pomace content caused the reduction of water activity at a similar level to pectin. This implies that infusing blackcurrant pomace at a certain level, due to its composition and interaction with water, may have a water activity-lowering effect as compared to that of pectin. Considering the association between water content and activity in the snacks and the amount of an additive, the LMP demonstrated extensive water-holding capacity compared to blackcurrant pomace powder making the water harder to remove, but not able to react. According to the related literature, the water holding capacity of dried blackcurrant pomace powder is over 10 times lower than pectin’s (Reißner et al. 2019), which confirms the obtained results.

**Table 2:** Physicochemical properties of the freeze-dried carrot-orange-ginger (COG) snacks obtained with dried blackcurrant pomace powder (BP) or low-methoxyl pectin (LMP): water content (WC), water activity (*a*_*w*_), hygroscopicity (H_72h_), hardness, porosity, and total color difference (*ΔE*)

Sample	WC (%)	*a*_*w*_	H_72h_ (g H_2_O/100 g)	Hardness (N)	Porosity (%)	*ΔE*
COG	1.92 ± 0.10^b^	0.055 ± 0.002^a^	29.15 ± 0.12^a^	21.98 ± 0.95^h^	59.50 ± 0.64^bc^	–
COG-BP1a	1.53 ± 0.10^c^	0.042 ± 0.001^b^	27.31 ± 0.12^b^	36.75 ± 3.10^g^	56.54 ± 0.69^cd^	19.52 ± 1.24^c^
COG-BP1b	1.48 ± 0.02^cd^	0.041 ± 0.002^b^	27.61 ± 0.34^b^	38.28 ± 2.45^g^	57.35 ± 0.44^d^	18.96 ± 0.98^cd^
COG-BP1c	1.38 ± 0.08^cde^	0.042 ± 0.002^b^	27.04 ± 0.41^b^	38.47 ± 1.41^g^	56.11 ± 1.31^d^	19.99 ± 1.27^c^
COG-BP3a	1.25 ± 0.08^e^	0.028 ± 0.001^d^	24.89 ± 0.19^c^	44.75 ± 2.68^f^	52.76 ± 0.43^e^	30.25 ± 1.14^b^
COG-BP3b	1.39 ± 0.08^cde^	0.029 ± 0.001^cd^	24.81 ± 0.16^c^	46.41 ± 2.27^ef^	52.64 ± 1.07^e^	30.94 ± 1.40^b^
COG-BP3c	1.28 ± 0.07^de^	0.028 ± 0.002^cd^	25.45 ± 0.37^c^	46.26 ± 2.27^ef^	51.53 ± 0.58^ef^	30.12 ± 1.34^b^
COG-BP5a	1.21 ± 0.01^e^	0.028 ± 0.002^cd^	22.90 ± 0.40^d^	49.39 ± 2.79^de^	49.15 ± 0.25^e^	35.73 ± 1.61^a^
COG-BP5b	1.19 ± 0.08^e^	0.024 ± 0.001^d^	22.76 ± 0.57^d^	53.23 ± 3.10^d^	49.85 ± 0.51^e^	35.34 ± 1.53^a^
COG-BP5c	1.27 ± 0.06^de^	0.027 ± 0.002^d^	22.41 ± 0.16^d^	59.42 ± 3.25^cd^	50.76 ± 0.27^ef^	35.41 ± 1.48^a^
COG-LMP0.5b	1.75 ± 0.01^b^	0.029 ± 0.003^cd^	27.60 ± 1.10^b^	79.60 ± 2.80^b^	61.57 ± 1.74^ab^	17.47 ± 2.34^d^
COG-LMP1.5b	2.15 ± 0.06^a^	0.034 ± 0.003^c^	27.56 ± 0.26^b^	142.99 ± 5.14^a^	62.10 ± 0.35^a^	19.28 ± 2.36^cd^

Different letters within columns indicate different homogenous groups determined by Tukey’s test at *P* < 0.05

Maintaining low water activity in food products is important in the aspect of microbial safety. Therefore, dehydration has become one of the most common food preservation methods. It has been established that the critical value of *a*_*w*,_ ensuring the food safety, equals 0.6 (Ijabadeniyi and Pillay 2017), which means that the results obtained for the snacks have been about ten times lower than the crucial parameter. However, water is also responsible for the glass transition (Silva-Espinoza et al. 2020a). Dehydrated products are often in an amorphous state that is manifested by hard and crispy texture, although, various dried products appearing on markets’ shelves prove that a lot of dried fruit and vegetables are dehydrated to the point in which water activity is reduced to the extent that ensures safety but not physical stability. Exceeded water content causes glass transition temperature decrease and, as a result, the food product becomes rubbery. In the case of freeze-dried products, the transition affects overall quality, such as texture, microstructure, and sensorial perception, taking away properties that differentiate these products from others (Moraga et al. 2011). For that reason, it is also important to examine the ability to absorb water from the environment, which for example indicates when the product should be consumed to avoid unfavorable changes in its physicochemical characteristics after the package opening.

The hygroscopicity was determined to check how prone the freeze-dried snacks are to absorb water, thus how high the risk of quality loss is. The main impellent of the mass change is the difference between the sample’s moisture content and environmental humidity, therefore, the samples characterised by low water content combined with open and porous structure usually reveal elevated hygroscopic properties. This may be considered a favourable feature in terms of rehydration but disadvantageous in the aspect of stability. The results obtained for the freeze-dried snacks exposed to the humid environment for 72 h are presented in Table 2. Initial moisture content in the samples varied from 1.12 to 2.00 g H_2_0/100 g d m., relative to the water content. As it can be seen, the additives significantly reduce water adsorption. The greater content of BP, the lower amount of water adsorbed by a snack. Pectin also limited sorption capacity, however, its effect was comparable to that observed for the lowest level of pomace powder (1%). Additionally, during the test, samples with the addition of the blackcurrant pomace obtained constant weight, thus the equilibrium moisture content, while snacks with LMP were still gaining water after 72 h. There was no difference in water gain for various amounts of added LMP, either, contrary to pomace powder, the increasing content of which was followed by decreasing hygroscopic properties. As a consequence, it is plausible to state that despite greater influence on water activity, pectin’s affinity for water (Panchev et al. 2010) limits its stability during storage, and so creates a demand for high-barrier packaging. Notwithstanding the reducing effect of BP addition, snacks obtained with it were also highly hygroscopic and hence should be isolated from humid environment as well. Hygroscopic properties strongly depend on the sample’s structure. Feng et al. (2022) suggested that a more dense and compact structure could inhibit water adsorption due to the superior amount of closed pores that are separated from the environment and thus harder to penetrate by humid air. On the other hand, the reduction of hygroscopicity resulting from pectin addition may have been caused by swelling of the hydrocolloid, and so closing accessibility of the internal pores (Kowalski et al. 2019). However, it also explains constantly growing moisture gain because of the water mobility and its transfer from the surface to inner parts of the sample.

### Microstructure and porosity

**Fig. 1:**
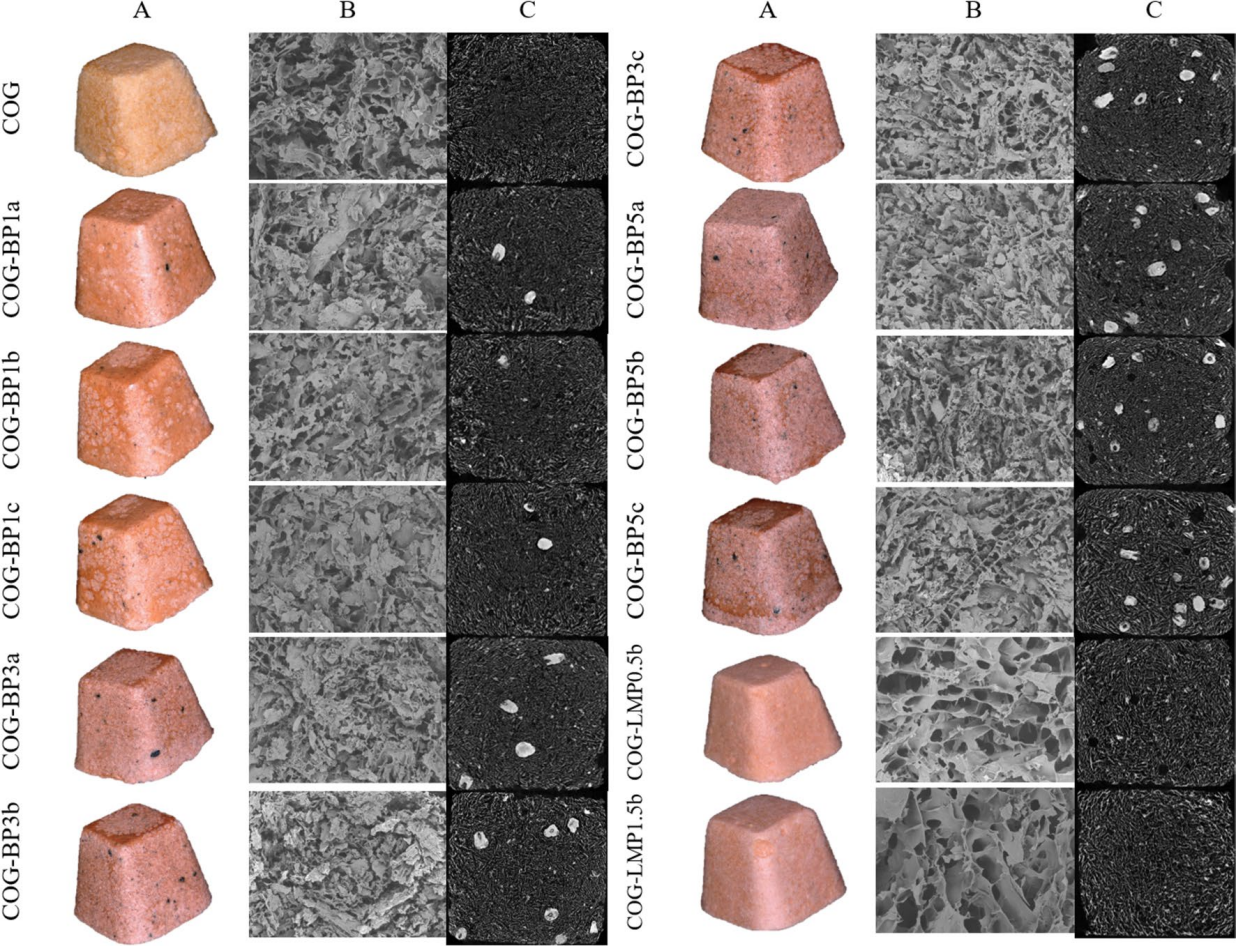
Photos of the overall appearance (section A), SEM imaging of the internal structure at magnification 200× (section B), and µCT reconstruction of the internal structure (section C) of freeze-dried carrot-orange-ginger (COG) snacks obtained with dried blackcurrant pomace powder (BP) or low-methoxyl pectin (LMP)

**Fig. 2:**
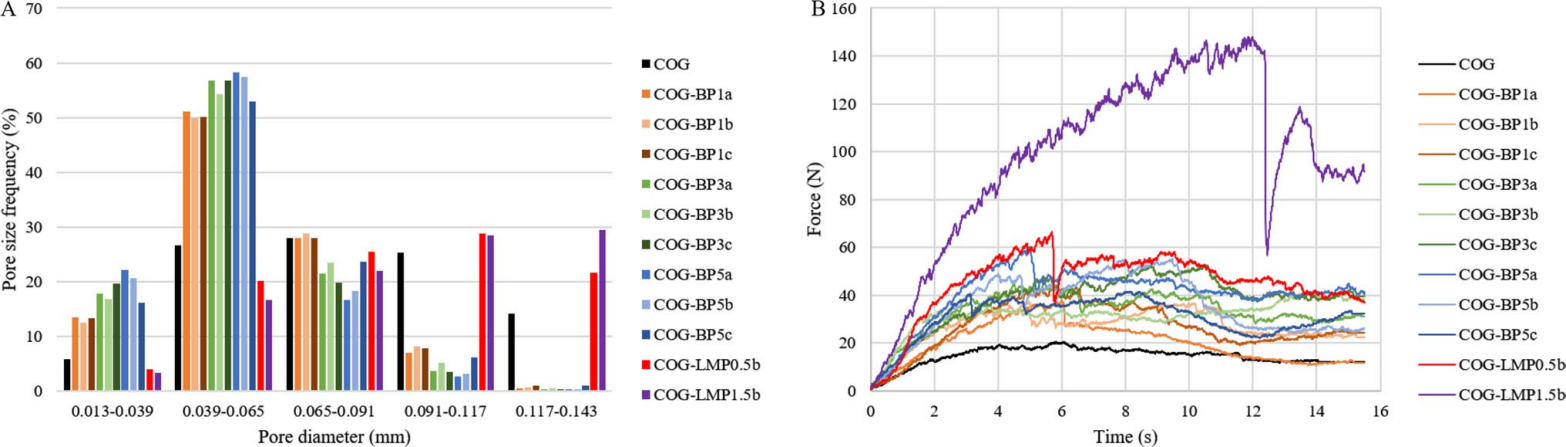
** a** Pore size distribution of freeze-dried carrot-orange-ginger (COG) snacks obtained with dried blackcurrant pomace powder (BP) or low-methoxyl pectin (LMP); **b** Exemplary compression curves of freeze-dried carrot-orange-ginger (COG) snacks obtained with dried blackcurrant pomace powder (BP) or low-methoxyl pectin (LMP)

Images of the internal structure of the produced snacks are presented in Fig. 1. Column A displays pictures obtained using scanning electron microscopy and column B shows a vertical cross-section developed by means of the computer-aided microtomography. As it can be seen, the type of used additive strongly determines the snacks’ structure. Samples with LMP additive featured relatively homogenous structures consisting of thin walls and large pores. On the other hand, a structure formed with BP was not as consistent and well-shaped as that with pectin. The pomace particles were explicit within the body of samples and pores were arranged unevenly. Moreover, the further towards the material’s centre, the more dense and compact structure occurred, which was also reflected in the higher frequency of small pores (Fig. 2a). Pores formation is determined by ice crystal growth, which depends on composition of the solid tissue. Pectin creates a hydrogel matrix featuring high flexibility, therefore crystals formulation due to freezing does not cause notable damage to the structure of the hydrocolloid network, and so the cellular structure can be maintained after freeze-drying (Feng et al. 2022). To enhance structure homogeneity in a hydrocolloid-stabilized matrix, ice crystal-controlling additives may be suggested for further investigation. Compared to pectin, even pomace powder subjected to hydrothermal treatment does not have the ability to create a three-dimensional network with thin walls and included air bubbles (Reißner et al. 2022), which explains the irregular structure of the snacks with BP.

The term porosity indicates the ratio of pore volume, free spaces in the sample structure, to the total volume of the sample. It determines product quality and its features related to e.g. mass diffusivity. In the case of dried products, pores formation relies on the mechanism of water removal, hence the dehydration method. The porosity of the freeze-dried snacks is presented in Table 2. Hydrocolloids are known for their structure-forming properties that allow for the production of highly porous materials, which, after subjecting to freeze-drying, simulate a cellular structure of, for example, fruit tissue. Therefore incorporation of pectin enlarges the total porosity of the sample, especially at the level of 1.5%, and supports the development of larger pores (Fig. 2a). The opposed effect was observed after BP addition. The pomace powder at 1% significantly reduced the porosity of the snacks and the extension of its amount led to a further decrease. Although the increasing amount of BP tended to be followed by the porosity reduction, the statistical analysis did not prove any significant difference between snacks with 3 and 5% of the pomace. However, as it can be seen in Fig. 2a, samples with a growing amount of BP in the formulation were characterised by a prevailing frequency of small-sized pores. In the beginning, fortifying the gelation process using calcium ions was expected to dictate porosity and microstructure characteristics but no significant impact was assessed in the aspect of porosity. It is not clear whether calcium infusion affected the size of the developed pores or not. The results obtained for porosity were consistent with the previous findings regarding freeze-dried multi-compound snacks but a much wider range of pores size was noted (Karwacka et al. 2022b). Porosity affects the sample’s hygroscopic properties, especially for the products containing high sugar content. However, in the case of freeze-dried guava pulp with pectin and sucrose, rising sugar content leads to water adsorption decrease due to the lowering porosity of the products, and so enhances stability during storage (Conceição et al. 2016).

### Mechanical properties

One of the main objectives of applying blackcurrant pomace to the formulation of freeze-dried snacks was to improve products’ features resilient to destroying factors affecting the sample, thus mechanical properties. The texture analysis helps better understand the product changes during processing, packaging, and consumption. The results of the mechanical properties study in terms of hardness that is the maximum force recorded during compression of the samples and exemplary compression curves are shown in Table 2; Fig. 2b, respectively. The lowest hardness characterised the initial COG sample, while the highest was recorded for the sample with pectin at 1.5% and then 0.5%. The gradual increase in the BP content resulted in the improved hardness of the snacks. As it was supposed, not only pomace powder was applied to strengthen the sample structure but also calcium ions should have supported the formation and consolidation of the structure (Byun et al. 2020). However, a meager upward tendency can be observed, especially between samples with 0.01% fortification and without any additive, the statistical analysis of the obtained results showing that changing concentration of calcium ions does not significantly influence hardness of the snacks.

When it comes to the course of material destruction, visible differences can be seen in the obtained curves (Fig. 2b). Peaks and drops on the curves portray the fracturing of the walls creating internal structure. Moreover, the more turbulent the curve, the more crispy the material. First of all, characteristic drops occurred on curves for snacks structured with pectin, which indicated some great cracks in the material structure that appeared resulting from the growing strain during compression. The course of COG-LMP1.5b compression was crucially distinguished from others in terms of the force range but its shape was similar to that obtained for COG-LMP0.5b regardless of the time in which the main cracks happened. On the other hand, samples without any additives were compressed accounting for the flattest and smooth curves, hence increasing BP content was followed by obtaining rougher curves, which proved rising crispiness. Although the course of the compression curves was changing for the samples with increasing amounts of structuring components, large drops that could be compared to those noted for the samples with pectin started occurring only for the snacks with the highest addition of BP (5%). However, there was no such an observation for each sample and the recorded differences in the force at the beginning and at the end of the drop were smaller. As it can be seen in Fig. 2b, existing drops and peaks were less rapid and less sharp as compared to the curves for the snacks with LMP. As suggested by Feng et al. (Feng et al. 2022), the oscillation of the compression force directly corresponds to microstructure characteristics. The more irregular collocation of pores, the rougher the curves. Moreover, the texture of the snacks is crucial and in the case of low-moisture products, it strongly depends on the water content, therefore, maintaining it at a low level provides crispy and brittle texture, which may be lost as a consequence of extensive water adsorption (Mazumder et al. 2007).

### Colour

**Fig. 3:**
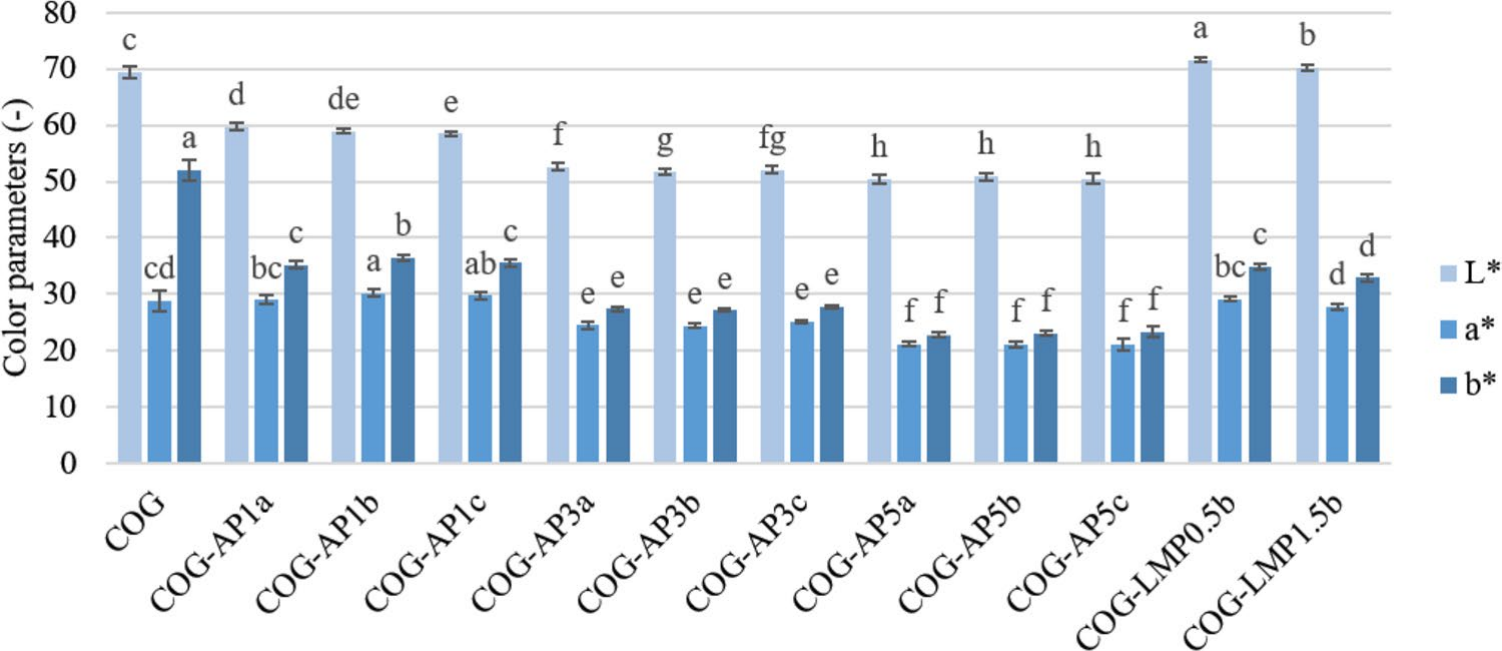
Color parameters L* (lightness), a* (redness) and b* (yellowness) of freeze-dried carrot-orange-ginger (COG) snacks obtained with dried blackcurrant pomace powder (BP) or low-methoxyl pectin (LMP). Results are presented as means with standard deviations. Different letters above bars in the same color indicate different homogenous groups determined by Tukey’s test at *P* < 0.05

The results gathered due to the instrumental analysis of the colour properties are presented in Fig. 3, and the total colour difference (*ΔE*) is shown in Table 2. Lightness (L*) increased after LMP incorporation but the addition of BP caused significant darkening of the snacks that was reflected in the lower values of the L* parameter. Chroma parameter a* indicating redness did not change in the samples with pectin as compared to the COG. However, the influence of pomace powder was inconsistent, and the 1% addition slightly heightened redness, while a further increase in the amount of BP notably lowered the a* parameter. The b* parameter portrayed yellowness when positive or blueness when negative. It can be seen that both of the used carriers caused a significant reduction in the yellowness. The effect of the lowest tested BP concentration was comparable to the one obtained by adding LMP, and having concurrently increased the concentration gradually kept decreasing b* values. The differences in the colour parameters measured for the snacks were also reflected in the total colour difference, which was evaluated in relation to the COG snack. The results were remarkably higher than 5, which was the value representing the changes that are hardly visually notable and as such imperceptible for a human eye. In order to show how the BP addition could influence consumers’ perception of the produced snacks, the images of all the collected samples have been included in Fig. 1. Similarly to the parameters mentioned above, *ΔE* caused by BP at 1% was corresponding to the one observed after pectin addition, however the increasing amount of pomace powder concurrently caused the colour difference to escalate. Similar results were expected due to the natural colour of the blackcurrant berries as well as blackcurrant pomace (Michalska et al. 2017a; Karwacka et al. 2022a), the pomace particles of which were occurring in the whole sample volume and on its surface. The colour results from all the ingredients infused into the products, and so the effect of the additives tested in this research is simply a consequence of using materials characterised by completely diverse features. The low-methoxyl pectin powder is light, whitish and creamy, while the blackcurrant pomace powder can be described as dark and deep burgundy (Karwacka et al. 2022a).

### Total polyphenols content

**Fig. 4:**
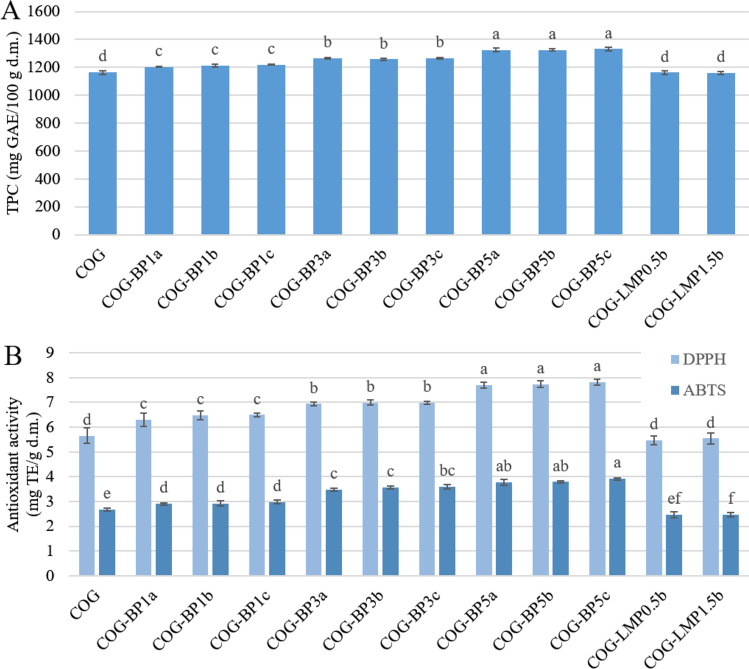
Total phenolic content (TPC) **a** and antioxidant activity against DPPH and ABTS **b** of freeze-dried carrot-orange-ginger (COG) snacks obtained with dried blackcurrant pomace powder (BP) or low-methoxyl pectin (LMP). Results are presented as means with standard deviations. Different letters above bars in the same color indicate different homogenous groups determined by Tukey’s test at *P* < 0.05

The total polyphenols content (TPC) in the freeze-dried snacks is presented in Fig. 4a. Its initial content in COG snacks was 1163.87 mg GAE/100 g d.m. The addition of LMP did not affect the TPC results but a notable trend related to an increasing amount of blackcurrant pomace was observed. The average growth of polyphenols content caused by the BP infusion at 1, 3, and 5% was estimated to equal 2.77, 3.68, and 4.90%, respectively. The relatively low effects probably resulted from the multi-step processing of the by-products. After juice production, pomace was dehydrated by means of the hot-air drying method, ground, which also induced heating of the material, then hydrated at elevated temperature and freeze-dried. Each stage of treatment could cause a significant loss of polyphenols due to thermal degradation or oxidation (Struck et al. 2016). Blackcurrant berries as well as blackcurrant pomace usually demonstrate high content of bioactive compounds, especially anthocyanins that also perform as antioxidants (Michalska et al. 2017b). Accordingly, a greater effect was expected to be achieved on the antiradical activity of the snacks.

### Antioxidant activity

Antioxidant activity was assessed as scavenging activity against two standard free radicals, and the obtained results expressed in terms of the Trolox equivalent are shown in Fig. 4b. Antioxidant activity of the initial sample without any additives equalled 5.66 ± 0.31 and 2.68 ± 0.07 mg Te/g d m., respectively for DPPH and ABTS assays. Correspondingly to the total polyphenols content, the snacks with LMP were not distinguished from COG samples in terms of antioxidant activity against DPPH but a slight reducing trend was observed in ABTS assay results. More importantly, the significant growth followed the addition of BP powder. When it comes to DPPH, the increasing amount of the added pomace powder induced the rise of antioxidant activity by 13.60, 23.26, 36.98% in the sequence following the level of addition, while ABTS results showed the tendency to enhance antiradical properties of the snacks by 9.78, 32.65, 44.65%. As mentioned above, blackcurrant pomace contains various bioactive compounds demonstrating antioxidant properties (Michalska et al. 2017a), therefore the antioxidant activity in result of the BP infusion was enhanced to the extent greater than could have been anticipated based on the TPC results. This outcome may also be supported by the previous findings that imply the enhancement of the antioxidant activity in result of the antiradical potential of derivative products arising from bioactive compounds degradation (Kruszewski et al. 2021).

## Conclusion

The findings of this study demonstrate that the incorporation of dried blackcurrant pomace powder (BP) has a beneficial impact on the physicochemical properties of the multicomponent freeze-dried snacks. As compared to low-methoxyl pectin, blackcurrant pomace has demonstrated lower texture and structure-forming ability, which has resulted in a more compact and irregular structure featuring a large number of small pores. The use of the additives has led to a significant reduction of hygroscopicity, which probably is the consequence of microstructure characteristics. The infusion of blackcurrant pomace powder significantly increases polyphenols content and extends the antiradical capacity of the products. However, the chemical properties become enhanced, and the anthocyanins added with the pomace cause significant colour changes including darkening of the snacks, which may not be evaluated as favourable. Taking into account the results obtained for the snack properties, it seems that the samples with 5% BP additive revealed the features corresponding to those demonstrated by the samples with 0.05% LMP additive to a greatest extent. The tendency regarding calcium ions concentration has been noted in terms of texture and structure but the effect has been insignificant. Accordingly, the lowest tested level can be recommended for further research, just to improve nutritional value and maintain established trends. But extensive research on the influence of calcium ions on the structure and texture-forming properties of blackcurrant pomace is also suggested. Although further research is needed, this study has shown another possibility for blackcurrant by-products management that had not been considered previously.

## Data Availability

Data will be available upon a reasonable request.
